# Study of the Influence of the Copper Component’s Shape on the Properties of the Friction Material Used in Brakes—Part One, Tribological Properties

**DOI:** 10.3390/ma16020749

**Published:** 2023-01-12

**Authors:** Andrzej Borawski

**Affiliations:** Faculty of Mechanical Engineering, Bialystok University of Technology, PL-15351 Bialystok, Poland; a.borawski@pb.edu.pl

**Keywords:** mechanical engineering, brake pads, friction, wear, tribological properties, ball-on-disc, ball-cratering

## Abstract

Brakes play an extremely important role in any vehicle. In today’s automotive industry, friction brakes are most often used, in which the composite material of the brake pad cooperates with a cast iron disc. While little can be modified in the case of discs, in the case of pads, the composition of the material used for its production can have an almost unlimited number of possibilities. Both scientists and manufacturers invent and test new combinations to achieve the desired end result. A similar task was undertaken in this work. Bearing in mind the fact that materials commonly used as reinforcing materials generate undesirable threats in the production process, it was decided to check whether this role could be taken over by another substance that is already present in brake pads; this substance is copper. A number of samples containing copper, in the form of powder and fibers, were made, and then the samples were subjected to tribological tests in order to determine the coefficient of friction and abrasive wear rate. The ball-cratering research method was used, and the Taguchi process optimization method was used to plan the experiment. As a result of the tests, it was found that the replacement of aramid fibers with copper fibers does not significantly affect the value of COF and the abrasive wear rate.

## 1. Introduction

Brake pads are very important elements of any vehicle. They have a direct impact on the effectiveness of the braking system, and thus on the braking distance. Therefore, the friction material of the pads is required to have long-term durability, stable properties, resistance to wear and weather conditions [1], and of course, ecological indifference [2].

The current technology allows the use of almost any available material to develop the desired composition of the friction material [3]. This composition may differ depending on the intended use of the pad (e.g., it will be different in racing vehicles and different in low-speed vehicles), but in virtually all cases four basic groups of components can be distinguished: reinforcements, binders, fillers, and abrasives [4]. Each of them performs a different task, and their proper proportions and the manufacturing process, which are often kept secret by the company, allow for a satisfactory final product.

However, scientific progress and health awareness resulted in a ban on the use of certain materials. One of the most famous banned materials is asbestos. Its use has been forbidden due to its carcinogenic properties [5]. In brake pads, it worked perfectly as reinforcement [6,7]. It is a fiber of natural origin, so its production did not cause unnecessary contamination. The process itself was uncomplicated and, therefore, cheap.

After asbestos was phased out, numerous studies were conducted to develop a substitute. Currently, synthetic materials are most often used as reinforcement, e.g., carbon fiber, aramid [8,9] or more exotic materials of natural origin, such as palm kernel shells [10], banana peels [11], periwinkle shells [12] or cocoa bean shells [13]. These materials are usually obtained as a by-product of other processes, e.g., food production, but they do not have good mechanical properties. Their use in the brake pads of motor vehicles, despite being eco-friendly, is, therefore, rather limited.

Synthetic materials, in turn, have excellent strength properties, but the process of their production involves the use of aggressive chemicals, is dangerous, or generates toxic waste [14].

Currently, aramid fibers are produced in the process of low-temperature polycondensation of para-phenylenediamine (PPD) and terephthaloyl chloride (TCL) monomers. PPD is a highly sensitizing aromatic amine, used, among others, for dyeing hair, fur and fabrics. The by-product of fabrication is hydrochloric acid. The production of aramid fibers and fabrics is expensive due to the difficulty of using the concentrated sulfuric acid, needed to maintain the water-insoluble polymer during synthesis and spinning [15,16].

Another example of a harmful material often used as reinforcement in friction materials is carbon fiber; in its production and processing there are three problem areas: dust inhalation, skin irritation and the effect of fibers on electrical devices. CF-coating materials often contain chemicals that can cause severe skin reactions, which also require protection [17,18].

One of most important components of brake pads is copper. Although it is harmful to humans [19], its excellent properties make it one of the mandatory components of each brake pad. In contact with the brake disc, due to friction, copper smears over the contact surface. This reduces the value of the coefficient of friction, which prevents overheating and reduces the abrasive wear rate [20]. In addition, the high thermal conductivity coefficient means that the energy generated in the braking process is effectively discharged from the friction node into the material. It turns out that the percentage of copper, as well as the size and shape of its particles, is also important [21,22].

Sellami et al. investigated the effect of both the particle size of copper alloys and their concentration. Samples with an average particle size of 80 and 500 µm were used. Their shape can be both solid with sharp edges, and spherical. Both the thermal and mechanical properties of the prepared samples were determined. This study found that larger particles have a positive effect on the mechanical properties, but do not change the thermal properties; they also cause instability in the coefficient of friction. According to the authors, large and irregular particles caused the layer of solid lubricant to form faster, thus affecting the values of the COF and the abrasive wear rate [23].

A similar topic was dealt with by Wojciechowski et al. The main difference was the geometry of the copper particles used in the tested samples. In this case, copper powder and fibers were used. The results of tribological tests showed that the fibrous form of copper gave a higher value for the friction coefficient and that it had a faster abrasive wear [24].

A completely different approach was presented by Lin et al. In research they checked the properties of completely copper-free materials, and compared the results with the standard composition of the friction material—NAO. They proposed six substitutes: coke, carbon black, carbon fiber, artificial graphite, natural graphite and expanded graphite. This study found that the carbonaceous components behave similarly to copper, creating a thin contact layer on the surface of the cooperation. However, the best choice was a specimen containing expanded graphite. It had the highest thermal conductivity, a stable coefficient of friction and a relatively low abrasive wear rate [25].

The influence of the percentage of copper content and its shape was also checked by Kumar and Bijwe. Copper, in the form of particles and fibers, was used. Samples containing 0, 10 and 20% of copper were made. They were characterized in terms of physical, thermophysical, chemical and mechanical properties. The higher content of copper in the form of powder gave better resistance to abrasive wear. However, there were no significant discrepancies in the coefficient of friction, which, in all tested cases, was between 0.35 and 0.45 [26].

Analyzing the above research results, it was decided that whether copper in the form of fibers can replace synthetic materials, used as a reinforcing material, would be checked. Such a material would not lose its valuable properties that copper gives it, and, at the same time, it would be much safer and less toxic in its production compared to classic compositions. The aim of this work is to investigate whether the use of copper fibers, as reinforcement, will affect the tribological properties of the friction material.

## 2. Materials and Methods

**Figure 1:**
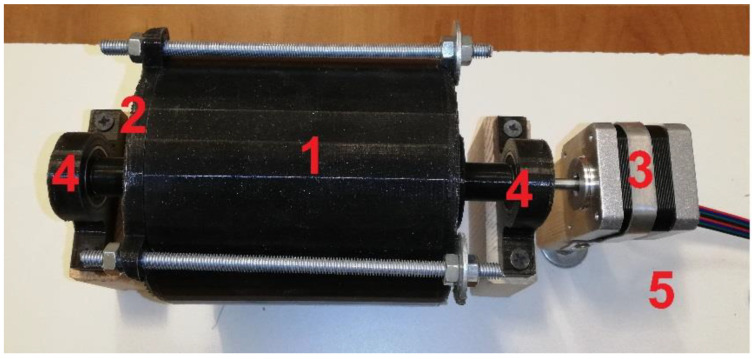
The 3D-printed mixer used in material preparation process: 1—container with mixing blades inside, 2—cover, 3—steeper motor (for precise rotation controlling), 4—bearing, 5—base.

For the tests, 4 series of samples, with the composition presented in Table 1, were made. In each group, 5 samples were prepared (1″ radius, 10 mm high), which allowed a reduction in the error of the production process; this would improve the repeatability of the results. Using a precise Steinberg SBS-LW-300A balance (accuracy 10^−3^ g), mixtures of ingredients were prepared in accordance with the following proportions (S1…S4). Then, each mixture was placed for one hour in a mixer (50 RPM), which was made in 3D printing technology for the purposes of this study (Figure 1).

The main difference between the individual sample groups was the following:

- in S1 samples, copper powder was used (grain size of <600 µm):
- in S2 samples, the copper was in the form of fibers with a length of 2–3 mm, and the aramid content was reduced by 4%;
- in S3 samples, the copper was in the form of fibers with a length of 4–6 mm, and reinforcement content was reduced by another 4%;
- in S4 samples, copper was in the form of fibers 7–10 mm long, aramid was not used, and copper fibers were only reinforcement.

**Figure 2:**
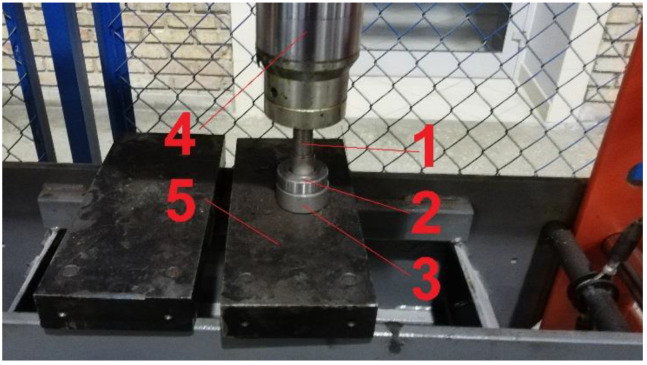
Forming sample process: 1—stamp, 2—form, 3—base, 4—hydraulic press, 5—press frame.

Prepared in this way, the mixture was placed in steel molds and compressed to a pressure of 20 MPa. A hydraulic press was used for this purpose (Figure 2).

**Figure 3:**
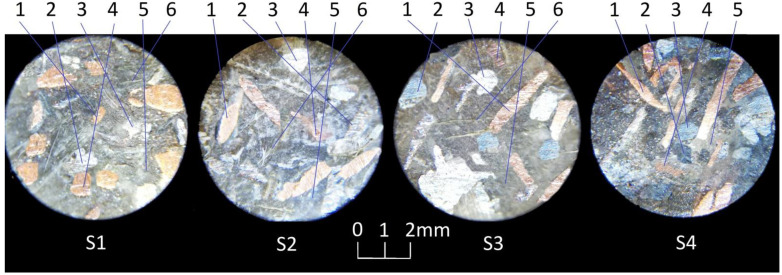
Micrograph of the surface of samples from each group: 1—copper, 2—cast iron, 3—steel, 4—brass, 5—matrix (resin with graphite and ash), 6—aramid.

After drying, the samples were ground using a magnetic grinder. In the research method that was used, it is important that the surface has the lowest possible roughness, preferably below 0.5 Ra; a value of ~0.32 was obtained for all samples (9th class). One sample of each group is presented in Figure 3.

**Figure 4:**
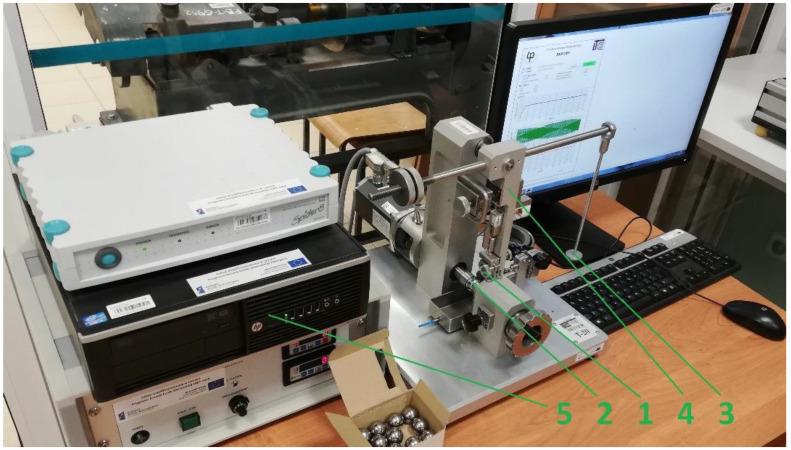
Picture of the T-20 test stand: 1—sample, 2—counter-sample (sphere), 3—load, 4—swivel leaver, 5—PC.

The tests were carried out on the T-20 test stand, which is provided by the Faculty of Mechanical Engineering of the Bialystok University of Technology. The picture of this stand is shown in Figure 4. The basic components are as follows: sample (1), counter-sample (2), a load ensuring the assumed contact pressure (3), a body with a rotary lever (4), and a computer with signal processing equipment (5). The counter-sample was a 1″ radius ball made of cast iron (3.1% C, 1.9% Si, 0.6% Mn). They were made in the zero accuracy class, and their hardness was 20 ± 2 HRC.

This method is successfully used in various fields of science [27,28,29,30,31]. Tests using ball contact are also performed, for example, in chambers with regulated conditions, e.g., temperature [32]. Counter-samples of various shapes are also used, e.g., a cylinder cooperating with a sphere with its side [33] or front surface [34]. Using it to study composite materials, e.g., friction materials, requires proper planning of the experiment. The Taguchi process optimization method was used for this purpose. Since 3 variables need to be determined, the orthogonal array will have 9 lines (Table 2).

In these studies, the recording frequency was set to 2 Hz. A new counter-sample was used for each test, and the cooperating elements were washed and degreased with a solvent. The tests for each group of samples were repeated five times.

The contact of two bodies always begins with the so-called running-in. This is the period from the beginning of the test until the value of the friction coefficient stabilizes [35]. These data were discarded each time, and the arithmetic mean was calculated using the remaining points. Then, using the Amontons–Coulomb friction law, which is described by the equation [36]:

(1) $$f_{ij}=\frac{{\bar{F}}_{ij}}{L}$$

where *f*—coefficient of friction *i* run of *j* series of samples (where *i*, *j* = 1…5), *F*—calculated average friction force, *L*—load, the values for the coefficients of friction for individual tests were determined. The average values obtained for individual preliminary tests are presented in Table 3.

**Figure 5:**
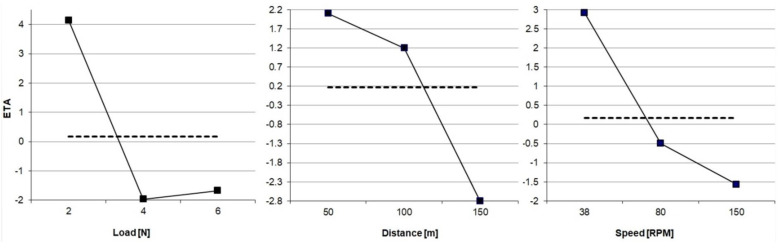
ETA function.

Then, referring to the criterion:

(2) $$\eta =-10log_{10}\left(\frac{1}{n}\overset{n}{\underset{i=1}{\sum }}y_{i}^{2}\right)$$

where *η* (ETA)—signal-to-noise ratio (S/N) function (Figure 5), *n*—number of measurements for a single sample (in this case repeated five times), *y*—result of a single test.

The determination of the above function required a total of forty-five preliminary tests, which allowed for the determination of the proper test parameters; these were the following: load—*L* = 4 N, distance—*S* = 150 m, and rotation speed—*n_r_* = 38 RPM. These parameters were used in the research, the results of which are presented below.

## 3. Results

Each sample was tested in triplicate for a total of 36 tests for all sample groups. As before, the running-in period was discarded in each study. The obtained results are summarized in Table 4.

These values made it possible to determine the coefficients of friction. Their arithmetic means and standard deviations were also determined according to the equation:

(3) $$S_{d}=\sqrt{\frac{\sum_{k=1}^{3}{\left(z-\bar{z}\right)}^{2}}{2}}$$

where *z*—average COF value of a single sample, and *k*—run no. Results are compiled in Table 5.

Another important aspect is the abrasive wear rate (*K_c_*). To determine this, the size of craters formed on the samples was used, and these were measured in two planes: in the direction of friction and perpendicular to it. Then, the average value for each of the measurements was determined; these values are summarized in Table 6. In addition, the standard deviation of each group was calculated.

**Figure 6:**
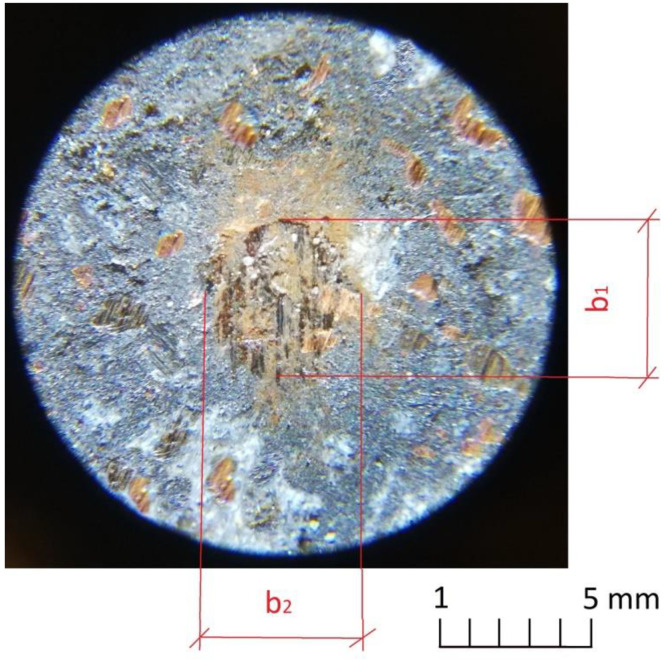
An example image of crater created during one of the preliminary test runs.

The above data allowed the determination of the abrasive wear rate. The Archard equation was used for this, which has the form [37,38,39]:

(4) $$K_{c}=\pi \frac{b^{4}}{64RSL}$$

where *R*—the counter-sample radius, and *b*—arithmetic average of the measurements of the crater diameters (for this purpose the Delta Optical microscope and Brinell magnifying glass were used, example on Figure 6):

(5) $$b=\frac{b_{1}+b_{2}}{2}$$

The results of calculations of *K_c_* for all four groups of samples are shown in Table 7.

## 4. Discussion

To properly analyze the obtained results for COF, the single factor analysis of variance method was used [40,41]. It compares the variability between the groups to the variability within the groups, and allows a comparison between the results of independent tests performed for at least three different groups.

It was assumed that the confidence level would be: α = 95%. Using the equation below, the number of degrees of freedom of individual groups was determined:

- for the qualitative factor:

(6) $$D_{fa}=a-1$$

- for random error:

(7) $$D_{fe}=N-a$$

- for total variation:

(8) $$D_{ft}=N-1$$

where *a*—the number of objects in the entire experiment, and *N*—the number of experimental units in the entire experiment. If the calculations were correct, the relationship below must be true:

(9) $$D_{fa}+D_{fe}=D_{ft}$$

The next step was to calculate the sum of squares based on the results of the experiment, using the formulas below:

- for the qualitative factor:

(10) $$SS_{a}=\overset{a}{\underset{i=1}{\sum }}n\left({\bar{x}}_{i}-\bar{x}\right)$$

- for random error:

(11) $$SS_{e}=\overset{a}{\underset{i=1}{\sum }}\overset{n_{i}}{\underset{j=1}{\sum }}\left(x_{ij}-{\bar{x}}_{i}\;\right)$$

- for total variation:

(12) $$SS_{t}=\overset{a}{\underset{i=1}{\sum }}\overset{n_{i}}{\underset{j=1}{\sum }}\left(x_{ij}-\bar{x}\;\right)$$

where *n*—number of repetitions, ${\bar{x}}_{i}\;$—object mean, $\bar{x}$—overall mean, and *x*—value of a single measurement for sample no.; there must be a relation between the *SS* values:

(13) $$SS_{a}+SS_{e}=SS_{t}$$

Mean squares calculations took the following form:

- for the qualitative factor:

(14) $$MS_{a}=SS_{a}/D_{fa}$$

- for random error:

(15) $$MS_{e}=SS_{e}/D_{fe}$$

The above calculations made it possible to determine the F-Fisher function values for each series of tests:

(16) $$F_{f}=\frac{MS_{a}}{MS_{e}}$$

From the statistical tables, taking into account the values calculated above and the *α* degree of confidence, the critical values for individual sample groups were read:

(17) $$F_{crit}=F_{\propto ,D_{fa},D_{fe}\;}$$

**Figure 7:**
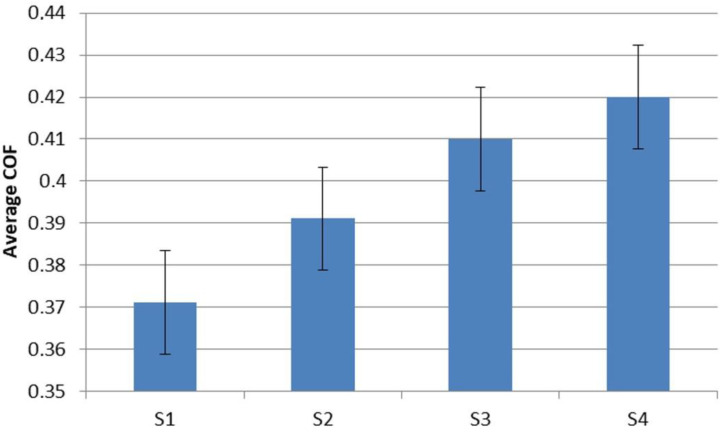
Expected marginal means, current effect: F(3, 32) = 16,024, p = 1543 × 10^−6^, decomposition of effective hypotheses, vertical bars represent 0.95 confidence interval.

The results of the above calculations are presented in Table 8 and in Figure 7.

A statistically significant influence of the material composition on the value of the coefficient of friction between the samples groups was found. This is confirmed by both the empirical *F_f_* values, calculated for each group of samples, satisfying, in each case, the relationship:

(18) $$F_{I\ldots IV}>F_{crit}$$

as well as *p*-values. Therefore, for the confidence level α, the zero hypothesis reads as follows:

(19) $$H_{0}:\;f_{1}=f_{2}=.\;.\;.=f_{4}$$

where *f*_(1…4)_—average COF value for each group of samples (I…IV), has been rejected. Therefore, in order to check the degree of homogeneity of variation in the groups, the Levene test [42] was used. The final test result describes the relationship:

(20) $$F_{Lev}=\frac{\sum_{i=1}^{a}n_{i}{\left({\bar{x}}_{i}-\bar{x}\right)}^{2}/\left(a-1\right)}{\sum_{i=1}^{a}\sum_{j=1}^{n_{i}}n_{i}{\left({\bar{x}}_{ij}-\bar{x}\;\right)}^{2}/\sum_{i=1}^{a}\left(n_{i}-1\right)}$$

This allowed the following results to be obtained (Table 9):

Analyzing the obtained results, it can be concluded that the measurements in groups I, II and IV do not differ statistically significantly from each other. For the level of significance:

(21) p=100−α

can be considered equal. This means that the individual samples of the above groups have almost identical tribological properties. The situation is different in group III. The Levene test showed that individual samples from this group gave unique results. This can be interpreted as a measurement error or sample production error, which resulted in a slight discrepancy in the results of the friction tests.

The reasons for the differences between the sample groups is, primarily, in the different aramid contents. This material is characterized by a low coefficient of friction and good mechanical properties [7]. Its lower content caused the value of the friction coefficient to slightly increase (by about 13%); this was particularly in the case of S4 samples, compared to samples from the S1 group. Similar results were also obtained by Bhane et al. In their research, the addition of aramid resulted in a decrease in the value of the friction coefficient by as much as half [43]. Reducing the content of aramid and the associated increase in the coefficient of friction also has its advantages. A higher coefficient of friction will contribute to a greater braking force at the same contact pressure, thanks to which the braking distance can be shortened.

In the second part of the research, an increase in the *K_c_* coefficient was also noted; this was by about 8% for the S4 samples, compared to the S1 samples. The reason for this, as before, is the change in the aramid content. Other researchers noticed even greater differences in the value of the abrasive wear rate [44,45]. This means that brake pads made of the S4 material will have a slightly shorter lifetime.

Further study of the properties of the proposed friction materials is planned. In the second part, the author wants to present the results of mechanical property tests, while in the third part, the results of simulation tests for the braking processes of a vehicle, equipped with brakes made of the developed materials, will be shown.

## 5. Summary and Conclusions

The paper presents tribological tests of four groups of samples used as friction material for vehicle brakes. The aim of the research was to check whether it is possible to make brake pads in a more ecological way, i.e., by replacing the popular reinforcing materials with copper in the form of fibers. The analysis of the research results allowed this study to determine the following:

- the complete replacement of aramid fibers with copper fibers increases the coefficient of friction by about 13%;
- the replacement also changes the value of the abrasive wear coefficient by approximately 8%.

The static analysis also showed that in the S3 group, there is a statistically significant discrepancy in the results between the individual samples. This is probably due to sample production or measurement error.

In order to obtain a full picture of the usefulness of the proposed materials, the performance of further empirical tests is planned; these tests will determine the mechanical properties (part 2) and model tests, and determine the course of the heating process (part 3).

## Figures and Tables

**Table 1:** Composition of individual groups of samples.

Component	Contents, %			
	S1	S2	S3	S4
Brass (CuZn20)	12	12	12	12
Copper (Cu)	25	25	25	25
Steel (0.18% C, 0.5% Si, 1.65% Mn, 0.05% P, 0.02% S, 0.08% Mo)	7	7	7	7
Aramid	12	8	4	0
Resin	17	17	17	17
Graphite (C)	5	5	5	5
Fly ash	18	22	26	30
Cast iron EN-GJS-400-12	4	4	4	4

**Table 2:** Orthogonal table listing the input parameters of the preliminary tests.

Preliminary Test No.	Load [N]	Distance [m]	Rotation Speed [RPM]
1	2	50	38
2	2	100	80
3	2	150	150
4	4	50	80
5	4	100	150
6	4	150	38
7	6	50	38
8	6	100	150
9	6	150	80

**Table 3:** Preliminary tests results.

Preliminary Test No.	Average Friction Force Value [N]:				
	1	2	3	4	5
1	0.39	0.37	0.40	0.41	0.38
2	0.53	0.56	0.58	0.53	0.58
3	1.06	1.09	1.18	1.18	1.15
4	0.91	1.26	1.19	1.17	1.22
5	1.41	1.45	1.31	1.37	1.31
6	1.28	1.11	1.35	1.45	1.33
7	0.86	1.28	1.14	1.38	1.06
8	0.85	0.86	0.84	0.71	0.82
9	1.93	1.85	1.88	1.86	1.91

**Table 4:** The results of the average friction force in individual tests.

Group No.	Sample No.	Average Friction Force Value [N]		
		Run No. 1	Run No. 2	Run No. 3
I	1	1.52	1.48	1.45
	2	1.45	1.52	1.46
	3	1.51	1.43	1.54
II	1	1.50	1.53	1.63
	2	1.60	1.55	1.55
	3	1.47	1.62	1.55
III	1	1.52	1.73	1.63
	2	1.62	1.68	1.62
	3	1.73	1.62	1.66
IV	1	1.60	1.57	1.72
	2	1.76	1.66	1.79
	3	1.57	1.78	1.71

**Table 5:** The results of the average COF in individual tests.

Group No.	Sample No.	COF Value			Average	Standard Deviation
		Run No. 1	Run No. 2	Run No. 3		
I	1	0.38	0.37	0.36	0.37	±0.009
	2	0.36	0.38	0.37		
	3	0.38	0.36	0.38		
II	1	0.38	0.38	0.41	0.39	±0.013
	2	0.40	0.39	0.39		
	3	0.37	0.41	0.39		
III	1	0.38	0.43	0.41	0.41	±0.016
	2	0.40	0.42	0.41		
	3	0.43	0.40	0.41		
IV	1	0.40	0.39	0.43	0.42	±0.021
	2	0.44	0.41	0.45		
	3	0.39	0.44	0.43		

**Table 6:** Crater diameters measured in individual samples.

Group No.	Sample No.	Average Crater Diameter of Single Sample [mm]	Average Crater Diameter of Sample Group [mm]	Standard Deviation
I	1	1.49	1.47	±0.020
	2	1.45		
	3	1.47		
II	1	1.48	1.48	±0.015
	2	1.46		
	3	1.49		
III	1	1.47	1.49	±0.015
	2	1.49		
	3	1.50		
IV	1	1.48	1.50	±0.020
	2	1.52		
	3	1.67		

**Table 7:** Calculated abrasive wear rate values.

Group No.	K_c_ [m^4^·m^−2^·N^−1^]
I	98.462 × 10^−14^
II	100.261 × 10^−14^
III	103.005 × 10^−14^
IV	107.702 × 10^−14^

**Table 8:** Single factor analysis of variance calculations results.

Source of Variation	*Df*	*SS*	*MS*	*F_f_*	*p*
Qualitative factor	3	12.58 × 10^−3^	4195 × 10^−3^	16,024	1543 × 10^−6^
Random error	32	8.38 × 10^−3^	2618 × 10^−4^		
Total	35	20.96 × 10^−3^			

**Table 9:** Levene test results.

	Samples Group			
	I	II	III	IV
*F_Lev_*	0.1818	0.1333	3.500	1.1351
*p*	0.8381	0.8776	0.0983	0.3818

## Data Availability

Data sharing not applicable.
